# TNF Tolerance in Monocytes and Macrophages: Characteristics and Molecular Mechanisms

**DOI:** 10.1155/2017/9570129

**Published:** 2017-11-08

**Authors:** René Huber, Rolf Bikker, Bastian Welz, Martin Christmann, Korbinian Brand

**Affiliations:** Institute of Clinical Chemistry, Hannover Medical School, Carl-Neuberg-Str. 1, 30625 Hannover, Germany

## Abstract

Tumor necrosis factor (TNF) tolerance in monocytes and macrophages means that preexposure to TNF reduces the sensitivity in these cells to a subsequent restimulation with this cytokine. Differential effects arise following preincubation with both low and high doses of TNF resulting in absolute as well as induction tolerance affecting specific immunologically relevant gene sets. In this review article, we summarize the relevance of TNF tolerance *in vivo* and the molecular mechanisms underlying these forms of tolerance including the role of transcription factors and signaling systems. In addition, the characteristics of cross-tolerance between TNF and lipopolysaccharide (LPS) as well as pathophysiological aspects of TNF tolerance are discussed. We conclude that TNF tolerance may represent a protective mechanism involved in the termination of inflammation and preventing excessive or prolonged inflammation. Otherwise, tolerance may also be a trigger of immune paralysis thus contributing to severe inflammatory diseases such as sepsis. An improved understanding of TNF tolerance will presumably facilitate the implementation of diagnostic or therapeutic approaches to more precisely assess and treat inflammation-related diseases.

## 1. Introduction

TNF is a potent proinflammatory master cytokine modulating inflammatory processes, and its rapid induction is fundamental for the orchestration of the immune response [1]. The application of TNF over a certain time period can result in a reduced sensitivity of cells, organs, or organisms towards a subsequent stimulation with the same cytokine, a phenomenon known as tolerance [2, 3]. TNF tolerance can be induced by a pretreatment of monocytes and macrophages with both low and high doses of TNF and can occur as absolute tolerance or induction tolerance following restimulation [3, 4]. In addition to pure TNF tolerance, several forms of TNF/LPS cross-tolerance have been described [5-7]. In this review, we will summarize the *in vivo* relevance of TNF tolerance as assessed in different animal models. In addition, we will refer to general features and the known molecular mechanisms underlying different forms of tolerance including the role of transcription factors, signaling systems, and receptors. Finally, the pathophysiological impact of TNF tolerance on both the resolution of inflammation and immune paralysis as well as potential diagnostic and therapeutic aspects targeting tolerance-associated mechanisms or molecules will be discussed.

## 2. TNF Tolerance *In Vivo*

TNF tolerance was first described in rats and mice on the basis of reduced physiological responsiveness towards a subsequent stimulation with TNF following a repeated application of sublethal doses of this cytokine within a certain time period [2, 8]. Monocytes and macrophages were early supposed to be important cellular mediators of TNF tolerance [2], and later experiments confirmed that assumption [3, 9] although other cell types (e.g., hepatocytes, cardiomyocytes, or epithelial cells) also proved to be prone to tolerization [10, 11]. *In vivo*, TNF tolerance is characterized by organo- and cytoprotective effects, as represented by the protection of tolerized mice against subsequent injections of normally lethal TNF doses [12]. Appropriately treated mice, rats, and guinea pigs are protected from inflammation-related symptoms such as fever [13, 14], gastrointestinal toxicity [15], liver injury [16], anorexia [17], hypertension, hypothermia, and lethality [18, 19]. Apoptosis, an important and common feature of inflammation-related diseases such as sepsis [20], is also suppressed in tolerant cells [16, 21]. In addition, disease-related alterations of physiological functions such as food intake normalized faster in TNF-tolerized mice than in control individuals [18, 22]. Since *in vitro* studies demonstrated in Hep G2 cells that TNF-associated cytotoxicity was more severe at fever-like temperatures, it has been speculated that TNF tolerance may be of importance for hepatic decompensation during febrile episodes [23]. In a sarcoma mouse model, it has been shown that the pretreatment with sublethal TNF doses may not only prevent the toxic effects of lethal doses of TNF but also reduce its antitumour effects, even when further increased doses were applied in tolerant mice [8]. An infection with adenoviruses can induce a tolerant-like state towards TNF, an effect which prevents LPS-induced mortality and liver injury/failure of the affected mice [24]. In addition, acquisition of TNF tolerance has been presumed in the case of malaria-infected mice, in which Plasmodium infection and released TNF did not result in perceivable symptoms of disease [25].

## 3. Molecular Characteristics

### 3.1. General Features of TNF Tolerance

**Figure 1:**
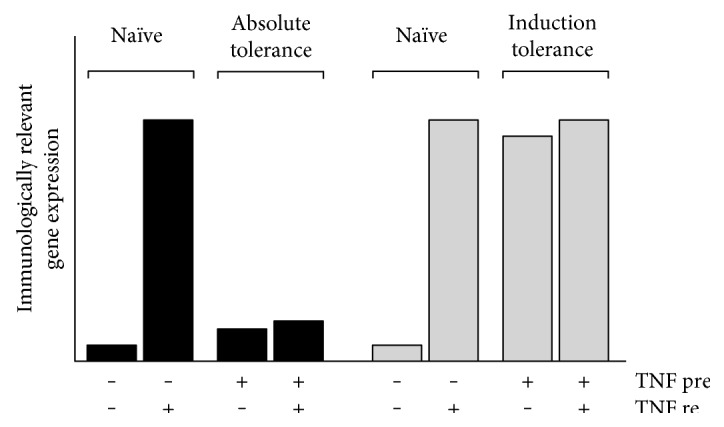
Two forms of TNF tolerance. TNF preincubation (pre) leads to reduced sensitivity towards further restimulation (re) with TNF (absolute tolerance) or causes elevated gene expression levels resistant to further upregulation following TNF restimulation (induction tolerance) in comparison to medium preincubated cells (naïve).

TNF tolerance can be induced by a pretreatment of monocytes and macrophages (or other eligible cells) with both low and high doses of TNF [3, 4, 26]. Typically, low-dose preexposure/incubation was performed using up to 10 *μ*g/kg (mostly human) TNF in animal experiments or up to 20 ng/ml in cell culture experiments [3, 27], while high doses included up to 100 *μ*g/kg in animal studies [2]. It also has to be taken into account that human TNF can induce tolerance in animal experiments using higher doses due to a reduced cytotoxicity, for example, in comparison to murine TNF which proved to be lethal at significantly lower doses [19]. Since the biological activity of different TNF samples and batches could vary significantly, TNF is latterly applied for cell culture studies in amounts standardized to the biological activity, that is, ≤40 U/ml for low and >40 U/ml for high TNF doses [4]. Following restimulation which is always performed using higher TNF concentrations, tolerance can be observed as absolute tolerance or induction tolerance. In absolute tolerance, a low expression of immunologically relevant genes can be found following TNF preincubation which remains on this level even in the case of a subsequent restimulation when compared to the short-term stimulation of naïve cells (Figure 1). Induction tolerance is characterized by an increased expression of genes following long-term preincubation with TNF which is in general roughly comparable with the level observed in naïve cells after short-term stimulation. Following TNF restimulation, the expression of these genes is “frozen” on this level and cannot be further induced (Figure 1). Gene groups affected by absolute and/or induction tolerance are related to cellular functions such as inflammation, growth/differentiation, chemotaxis/migration, signaling/transcription, and metabolism [4]. For instance, genes such as TNF, interleukin (IL-) 1*β*, IL-6, IL-8, or tissue factor are affected by absolute tolerance, whereas IL-18, IL-28A, IL-32, or toll-like receptor (TLR) 2 are prone to induction tolerance. Although TNF is normally applied for 48–72 h during preincubation [4, 26], a time period of 18 to 24 h has been generally described to be minimally required for the induction of TNF tolerance [5, 12] and enables the maintenance of the tolerized state for several days [12]. However, under certain conditions, even a TNF pretreatment for 2 h appears to be sufficient to induce protection from TNF-dependent cell/organ damage [21]. The signaling network involved in development, consolidation, and regulation of monocytic/macrophagic TNF tolerance is only partially investigated, and only a limited number of cell culture studies characterizing the molecular basis of TNF tolerance exist yet. Currently, it is realized that TNF tolerance is based on a variety of distinct but connected and mutually depending molecular events covering several regulatory spectra of intracellular signal transduction. In the following, the known molecular mechanisms determining TNF tolerance are discussed.

### 3.2. Low-Dose-Induced Tolerance

**Figure 2:**
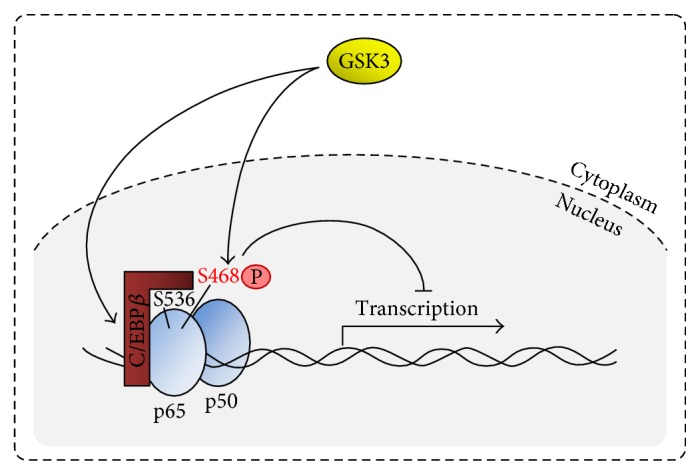
Transcriptional repression during low-dose-induced TNF tolerance. In low-dose TNF preincubated cells, p65 phosphorylation of the activating phosphorylation site Ser536 is blocked via direct protein-protein interaction with C/EBP*β*. In addition, an increased phosphorylation of the inhibitory site p65-Ser468 can be observed. Both events may be influenced by GSK3 and negatively regulate the transcription of immunologically relevant genes in low-dose TNF-tolerant cells.

Long-term preincubation with low doses of TNF predominantly leads to the development of absolute tolerance [4]. The expression of most of the genes suppressed under these conditions is regulated by the transcription factors nuclear factor *κ*B (NF-*κ*B) and/or activator protein (AP-) 1 [4, 28] indicating that their limitation is an essential step in restricting the expression of these tolerance-sensitive genes. In low-dose-tolerized and restimulated monocytic cells, I*κ*B*α* proteolysis, nuclear translocation of p65, and NF-*κ*B DNA binding activity are only weakly affected [26]. However, following restimulation of low-dose-tolerized cells, activation of IL-8 promoter- and *κ*B-dependent transcription is inhibited and p65 phosphorylation at the activating site Ser536 is markedly reduced in murine macrophage-like and monocytic THP-1 cells (“Tohoku Hospital Pediatrics-1”, [29]) [4, 9] (Figure 2). The latter effect may be ascribed to an increased association of p65 with the transcription factor CCAAT/enhancer binding protein (C/EBP) *β* in low-dose-tolerized cells [9]. In this context, protein-protein interaction of p65 and C/EBP*β*, which is an important regulator of proliferation and differentiation in myelomonocytic cells [30], results in a blockade of p65-Ser536 (and possibly other activating phosphorylation sites) [9, 31]. In addition, under these conditions (but also in high-dose-tolerized cells), p65 phosphorylation is intensified at Ser468 [4], a phosphorylation site negatively regulating p65 activity [32]. Interestingly, p65-Ser536 phosphorylation is resumed and low-dose TNF-induced tolerance is reversed by glycogen synthesis kinase (GSK) 3 inhibition using SB6763 [4], an effect also described within TNF-induced cross-tolerance towards LPS [6]. Furthermore, it has been reported that GSK3 phosphorylates C/EBP*β* [33] as well as p65-Ser468 [32] which suggests a multistep influence of this kinase in TNF tolerance.

Moreover, both low- and high-dose TNF-preincubated THP-1 cells are characterized by attenuated phosphorylation of c-Jun N-terminal kinase (JNK), extracellular signal-regulated kinase (ERK), and p38 [4, 5], that is, kinases targeting (amongst others) AP-1 subunits of the Jun and Fos protein families [34]. Total levels of these kinases, however, appear not to be significantly affected [4, 5]. As a consequence, concentrations of phosphorylated c-Jun were considerably lower in tolerized and restimulated monocytic cells than in stimulated naïve cells [4].

In murine models, it has been shown that a blockade of the glucocorticoid receptor (GR) using the antagonist RU-38486 can prevent the occurrence of low-dose-induced TNF tolerance *in vivo* suggesting that glucocorticoids and their receptor(s) contribute to the formation of tolerance [19]. This might be due to GR-dependent functions such as inhibition of proinflammatory signaling pathways, reduction of AP-1 DNA binding and NF-*κ*B translocation, and induction of anti-inflammatory (e.g., IL-10) as well as downregulation of proinflammatory cytokine/chemokine (e.g., IL-6, IL-8) expression [35]. For other transcription factors, a role within TNF tolerance has not been established yet. TNF-inducible factors such as activating transcription factor (ATF) or suppressor of cytokine signaling (SOCS-) 1, 2, and 3 have also been discussed with respect to an association with TNF tolerance [21]. However, ATF has not been analysed yet and for SOCS1-3, no influence on tolerance formation could be shown [21].

Taken together, the reported findings indicate that low-dose TNF-induced absolute tolerance is predominantly controlled via transcriptional mechanisms, that is, C/EBP*β*-dependent suppression of p65 phosphorylation and potential GSK3 activity (Figure 2). In addition, reduced c-Jun/AP-1 activation and GR-dependent transcriptional repression may contribute to the development of low-dose-induced tolerance.

### 3.3. High-Dose-Induced Tolerance

Long-term preincubation with high TNF concentrations may result in the development of both absolute and induction tolerance [4]. Following restimulation, nuclear levels of p65 and NF-*κ*B DNA binding activity are significantly reduced in high-dose-tolerized monocytic cells [4] in comparison to naïve and low-dose-tolerized cells [26]. In cells, long-term pretreated with high-dose TNF, I*κ*B*α* turnover is increased and I*κ*B*α* amounts are reduced to a lower level [4]. Following restimulation, however, no further stimulus-induced proteolysis of I*κ*B*α* can be observed. Consistently, it has also been observed that I*κ*B kinase (IKK) phosphorylation is completely inhibited in high-dose pretreated and restimulated cells when compared to naïve cells [4, 36]. In TNF-overexpressing murine cardiomyocytes, additional activation of p50 homodimers has also been found in comparison to control cells and it was speculated that this effect might represent an adaptive response to reduce the detrimental inflammatory consequences of the permanent presence of TNF [11].

**Figure 3:**
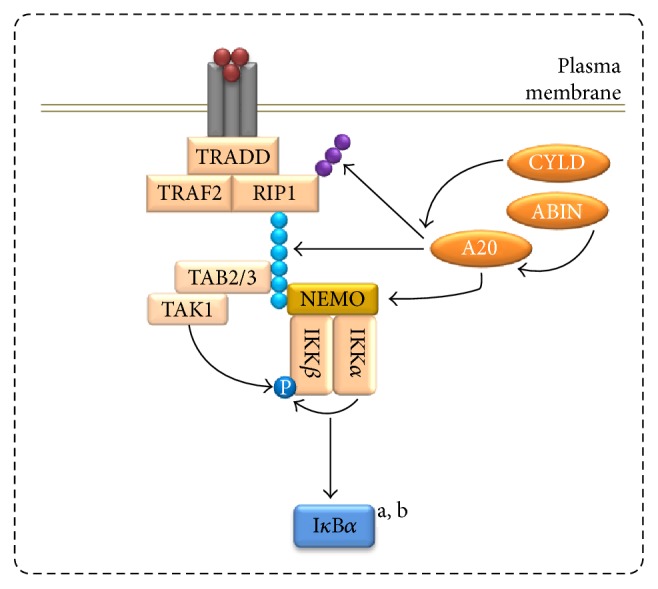
Inhibition of NF-*κ*B-associated signaling during high-dose-induced TNF tolerance. The signaling complex at the TNFR1 consists of tumor necrosis factor receptor type 1-associated DEATH domain protein (TRADD), TNF receptor-associated factor 2 (TRAF2), and RIP1. Via RIP-associated K63-linked polyubiquitin chains (blue dots), other proteins are recruited, especially the IKK complex (consisting of NF-*κ*B essential modulator (NEMO), IKK*α*, and IKK*β*) and the TGF-*β*-activated kinase (TAK) 1/TAK 1 binding protein (TAB) 2/3 complex. In high-dose TNF preincubated cells, IKK phosphorylation is inhibited by A20 presumably via noncatalytic binding of NEMO, induction of RIP degradation by K48-polyubiquitination (purple dots), and/or hydrolyzation of K63 polyubiquitins on several signaling proteins. A20-mediated restriction of IKK activity is supported by ABIN1 and CYLD and results in modulated I*κ*B*α* proteolysis: a, during pre-incubation, an increased I*κ*B*α* turnover leads to lower I*κ*B*α* levels; b, following restimulation, no further decrease of I*κ*B*α* levels occurs.

Due to the observation that A20 is a key molecule in the establishment of TNF/LPS cross-tolerance [6], its importance within TNF tolerance has also been assessed. A20 is a ubiquitinase/deubiquitinase known for its role as a repressor of NF-*κ*B signaling [37, 38] and characterized by three major functionalities, that is, noncatalytic mechanisms mediating the repression of IKK activation, ubiquitin ligase activity leading to K48-labeling of proteins such as receptor interacting protein (RIP) to induce their proteasomal degradation, and protease activity towards K63 and M1 polyubiquitins [39]. A20 mRNA and protein amounts are markedly upregulated in THP-1 cells as well as primary human monocytes and macrophages pretreated with high TNF doses [4, 36], an effect equivalently occurring in cross-tolerance [6]. The siRNA-dependent knockdown of A20 leads to a strong upregulation of IKK phosphorylation and proinflammatory gene expression in monocytic cells in which TNF tolerance was induced by a high TNF dose [4, 36] indicating that A20 is a major regulator of high-dose-induced tolerance (Figure 3).

A20 interacts and cooperates with additional factors to form the A20 ubiquitin-editing complex, that is, the adaptor molecules Tax1-binding protein 1 (TAX1BP1) and RING-finger protein (RNF) 11 [40, 41] as well as the E3 ubiquitin protein-ligase Itchy homolog (Itch) [42] which have been described to support A20 during the regulation of ubiquitin-dependent TNF signaling [43]. Due to their essential contribution to A20 activity, these three proteins appear to be promising candidates in the initiation of TNF tolerance, but neither TAX1BP1 nor RNF11 or Itch was significantly regulated on the mRNA level in monocytic cells incubated with the high TNF dose for 48 h [4, 36]. Beyond initial mRNA expression analyses, however, an involvement of TAX1BP1, RNF11, and Itch in TNF tolerance has not been addressed yet and remains to be established. In contrast, mRNA and protein expression of two other regulators cooperating with A20 is increased in high-dose TNF pretreated monocytic cells [36]: A20 binding inhibitor of NF-*κ*B (ABIN) 1, an A20 adjuvant protein [44], and cylindromatosis susceptibility gene (CYLD), a deubiquitinase possessing activities partly overlapping with A20 [39]. ABIN1 knockdown induced a modest elevation in IL-8 mRNA in TNF long-term-incubated cells which also could not be further elevated by TNF restimulation [36] indicating that ABIN1 cooperates with A20 in mediating TNF tolerance (Figure 3). CYLD knockdown results in an elevation of IL-8 mRNA in TNF long-term preincubated cells which was not further increased when the cells were restimulated with TNF. Effects of A20-siRNA application can be slightly further enhanced using a combination of A20- and CYLD-siRNA [36] suggesting that CYLD contributes to the A20-induced development of TNF tolerance and provides a certain amount of additive effects (Figure 3).

It has also been demonstrated in high-dose-tolerized primary human monocytes that protein phosphorylation is affected by protein phosphatase (PP) 1, an enzyme [45] characterized under these conditions by increased mRNA expression of the catalytic subunit PP1CB and downregulation of the regulatory subunit PP1R14C. Repression of PP1 activity by PP1R14C overexpression or calyculin A treatment resulted in an abolition of high-dose TNF-induced absolute tolerance [4].

Together, these data suggest a model in which high-dose TNF-induced tolerance especially depends on the suppression of NF-*κ*B-associated signaling by A20 which is supported by CYLD and ABIN1 (Figure 3). In addition, phosphatases may also be involved in the formation of TNF tolerance by reducing the global phosphorylation level in TNF preincubated cells.

### 3.4. Additional Mechanisms

In murine models, TNF tolerance can be induced by human TNF which acts as a selective inducer of murine TNFR1 but not murine TNFR2 [19]. Moreover, tolerance can be induced in TNFR2 knockout mice [16, 21] and the expression of glucocorticoids which may be involved in low-dose-induced TNF tolerance is activated by TNFR1-dependent signaling [19]. Thus, TNF tolerance appears to be mediated via TNFR1 [16, 19]. While total amounts of TNFR1 were not significantly affected during long-term treatment in monocytic THP-1 (high-dose TNF) or SW480 epithelial cells (low-dose TNF) [27, 36], TNF stimulation led to an internalization of the receptor within 2 h irrespective of the dose used [36]. Application of low-dose TNF during the preincubation phase, however, resulted in a (slow) recurrence of the initial level of TNFR1 within 48 h. In contrast, the use of high-dose TNF led to a permanent reduction of the receptor on the cell surface during the entire preincubation period, which could be reversed within 24 h when TNF was removed from the medium [36]. This suggests that receptor scarcity at the cell surface is another point restricting TNF-dependent signaling in tolerant cells, at least in primary human monocytes, since TNF pretreatment did not result in the downregulation of TNFR1 at the surface of tolerant murine hepatocytes [16]. However, the major functional role within the establishment of high-dose-induced TNF tolerance appears to be mediated by A20 as discussed above (see Section 3.3).

In mice and primary murine hepatocytes, low-dose TNF preexposure yields decreased amounts of hepatic ubiquitin-specific protease (USP) 2 [10]. Downregulation of USP2, either induced by TNF treatment or artificially using siRNA, resulted in the prevention of TNF-induced apoptosis, an effect occurring in combination with increased levels of cellular FLICE-like inhibitory protein (cFLIP), an antiapoptotic molecule [46], and downregulation of Itch [10], which acts as a cFLIP inhibitor in that context [47]. Vice versa, USP2 overexpression inhibited the establishment of TNF tolerance [10] indicating that USP2 represents a powerful inhibitor of tolerization, at least in the murine hepatic system.

## 4. Cross-Tolerance

The TNF-tolerant state may further include the refractoriness towards other stimuli. For instance, cells or animals tolerized with TNF are cross-tolerant towards gram-negative bacteria [7, 18], LPS [2, 6, 27], or other bacteria-derived agents such as lipophilic outer membrane vesicles [7]. Vice versa pretreatment with LPS [5, 48] or macrophage-activating lipopeptide 2 [49] induced the development of tolerance towards subsequent TNF application in THP-1 cells or mice and rats. Interestingly, tolerance may also be a result of certain infections, since adenoviral infection of mice has been described to yield a tolerance-like condition towards TNF treatment [24].

Due to the equivalent conditions they are creating especially in monocytes and macrophages, TNF tolerance and cross-tolerance appear to be based in part on overlapping or at least similarly operating molecular mechanisms. This assumption is already well substantiated in the literature revealing that both TNF- and LPS-induced tolerance/cross-tolerance are characterized by (1) decreased NF-*κ*B activity in the nucleus under certain conditions [4, 50], (2) GR-dependent gene regulation [19, 51], (3) attenuated mitogen-activated protein kinase phosphorylation [4, 5], (4) regulatory influence of GSK3 activity [4, 6], (5) upregulation of A20 [6, 36], and (6) receptor downregulation at the cell surface [36, 51]. Together, these events finally lead to an inhibition of (primarily NF-*κ*B-dependent) proinflammatory gene expression and the manifestation of a widely inert cellular state. However, since TNF tolerance and LPS/TNF cross-tolerance are established via different receptors (TNFR1 versus TLR4) [19, 50] and TNF tolerance is induced less rapidly by TNF than by LPS [22], specific mechanisms (depending on the respective inducer) influencing the unique features of both phenomena also have to exist.

Moreover, it has been described that the application of further agents can positively or negatively modulate the occurrence of TNF tolerance. For instance, the combined treatment of mice with TNF and leukemia inhibitory factor results in a significantly increased protective effect against a (generally lethal) LPS dose when compared to TNF application alone [52]. In contrast, the addition of human IL-1 completely prevented the development of TNF tolerance in mice normally induced by human TNF [19]. This indicates that an interaction or cross-reaction with other signaling pathways and regulatory events may modulate the events determining the occurrence of tolerance.

## 5. Pathophysiological Aspects of TNF Tolerance

To prevent deleterious consequences of inflammatory events such as excessive or chronic inflammation, a strictly controlled and fine-tuned termination of inflammatory processes is required [53-55]. As illustrated in this review, different forms of TNF tolerance can be observed following long-term (>24 h) preexposure to TNF [4, 5, 26]. The signaling quality occurring during the development of TNF tolerance significantly differs from the massive temporary activation of TNF-dependent signaling within the first 12 h of stimulation which has been characterized extensively [1, 56] and designated as phases I and II of TNF signaling [57]. Thus, TNF tolerance may play a role as a mechanism mediating refractoriness of monocytes and macrophages towards sustained proinflammatory cell activation in phase III (>12 h) of cytokine stimulation [36].

**Figure 4:**
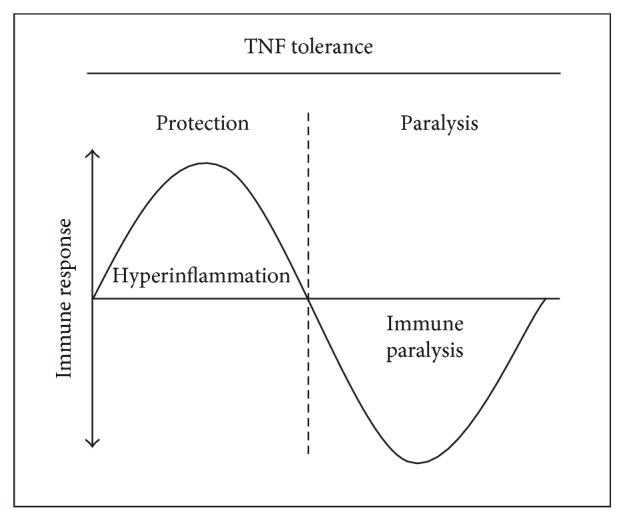
Two faces of TNF tolerance. To prevent hyperinflammation, refractoriness towards TNF may serve as a protective mechanism for cells and organisms to resolve inflammation and prevent acute or chronic inflammatory disease. On the other hand, excessive TNF tolerance may result in the paralysis of cellular immune functions, thus contributing to the state of immune paralysis characteristic for severe inflammatory diseases such as sepsis.

Due to its immunomodulatory effects, TNF tolerance may comprise several clinical implications in inflammatory and malignant diseases and represents a “Jekyll and Hyde”-like cellular process. On the one hand, tolerance may be a beneficial mechanism contributing to the resolution of inflammation and the protection from sepsis-associated hyperinflammation or prolonged chronic inflammatory diseases. In this context, loss of tolerance may favour the formation of chronic TNF-dependent inflammatory diseases in which NF-*κ*B is activated such as rheumatoid arthritis [58], inflammatory bowel disease [59], or autoimmunity [60]. On the other hand, “too much tolerance” may be regarded as a deleterious event presumably involved in the development of immune paralysis and the shutdown of the immune system observed during certain phases of sepsis [61-63] (Figure 4). The balance between protection against excessive inflammation and immune paralysis may finally determine a patient's fate [62]. In malignant diseases, refractoriness towards TNF may also play a role [64]. For instance, it has been observed that tumour formation may occur as an adverse event of anti-TNF treatment in several inflammatory diseases [1].

## 6. Conclusions

As summarized in this review, TNF tolerance is generally characterized by the inability of affected cells, organs, or organisms to fully respond to a restimulation with TNF following a preceding long-term incubation with the same stimulus. Thus, TNF tolerance may play a role in severe acute or chronic inflammatory diseases in which TNF is present in large amounts. As such, TNF tolerance appears to be an interesting target within the efforts to improve the therapeutic modulation of exaggerated and/or prolonged dysregulated immune responses. However, despite a variety of experimental approaches and considerable progress in the characterization of its molecular regulation in recent years, the complex network of mechanisms determining occurrence and establishment of TNF tolerance is not completely elucidated yet. In addition, the distinct role of TNF tolerance in development and progression of inflammation-related diseases as well as its clinical relevance has to be precisely established [65].

A better characterization of the mechanisms determining TNF tolerance will improve the understanding of its clinical relevance and presumably facilitate the development of diagnostic approaches to assess different forms and states of tolerance. In addition, increased knowledge on TNF tolerance potentially offers the implementation of therapeutic approaches to treat inflammation-related diseases either by initiating or breaking the establishment of TNF tolerance.

## Abbreviations

ABIN: A20 binding inhibitor of NF-*κ*B
AP-1: Activator protein 1
ATF: Activating transcription factor
C/EBP: CCAAT/enhancer binding protein
cFLIP: Cellular FLICE-like inhibitory protein
CYLD: Cylindromatosis susceptibility gene
ERK: Extracellular signal-regulated kinase
GSK: Glycogen synthesis kinase
GR: Glucocorticoid receptor
I*κ*B: Inhibitor of (NF-)*κ*B
IKK: I*κ*B kinase
IL: Interleukin
Itch: E3 ubiquitin protein-ligase Itchy homolog
JNK: Jun N-terminal kinase
LPS: Lipopolysaccharide
NF-*κ*B: Nuclear factor *κ*B
PP: Protein phosphatase
RIP: Receptor interacting protein
RNF11: RING-finger protein 11
SOCS: Suppressor of cytokine signaling
TAX1BP1: Tax1-binding protein 1
THP-1: Tohoku Hospital Pediatrics-1
TLR: Toll-like receptor
TNF: Tumor necrosis factor
TNFR: Tumor necrosis factor receptor
USP: Ubiquitin-specific protease.
